# SHIELDS OF CHIFFON: THE FORTITUDE/RESILIENCE OF AGING DRAG ARTISTS

**DOI:** 10.1093/geroni/igae098.0745

**Published:** 2024-12-31

**Authors:** Brian Chapman, Alaina Brenick, Laura Donorfio

**Affiliations:** University of Connecticut, Waterbury, Connecticut, United States; University of Connecticut, Hartford, Connecticut, United States; University of Connecticut, Waterbury, Connecticut, United States

## Abstract

This presentation discusses findings from a mixed methods study examining the intersection of aging, drag artistry, and individual wellbeing. Drag artists remain an understudied and underrepresented population in scholarship (Knutson et al., 2020; O’Brien, 2018). More specifically, older drag artists have yet to be studied in social science literature (Henneberry et al., 2022). Participants completed a semi-structured interview and a self-administered survey to understand the perspectives of drag artists over the age of 50 (n=22; ages 50-94). Qualitative analysis of the interviews revealed themes related to personhood, family impact, coping, resilience, generativity, community advocacy, activism, and performance. Participant interviews were augmented through an electronic survey that validated themes and provided a greater understanding of personality characteristics illuminated in the interviews. The survey assessed resilience (CD-RISC; Connor & Davidson, 2003), depression (CES-D; Radloff, 1977), personality (TIPI; Gosling et al, 2003), and coping (Brief COPE, Carter, 1997) of 20 drag artists (51-94 years). Descriptive analyses reveal high levels of resiliency (M =80.32/100) and low levels of depression (80% of participants reported below threshold rates/symptomatology). The sample is significantly more extraverted, conscientious, and open than population comparison means (51+ years) (t-tests; p’s=0.01-0.001). Repeated measures ANOVA Additionally, results showed that participants used healthier coping methods (i.e., problem-focused>emotion-focused>avoidant (Repeated Measures ANOVA, p’s<0.001). The lived experiences revealed in this study also provides data about the unifying impact of shared episodic events. The session will close with a discussion of potential future research including intergenerational approaches.

